# Extended Daily Dialysis in Acute Kidney Injury Patients: Metabolic and Fluid Control and Risk Factors for Death

**DOI:** 10.1371/journal.pone.0081697

**Published:** 2013-12-11

**Authors:** Daniela Ponce, Juliana Maria Gera Abrão, Bianca Ballarin Albino, André Luis Balbi

**Affiliations:** University São Paulo State-UNESP, Distrito de Rubiao Junior, Botucatu, São Paulo, Brazil; D′or Institute of Research and Education, Brazil

## Abstract

Intermittent hemodialysis (IHD) and continuous renal replacement therapies (CRRT) are used as Acute Kidney Injury (AKI) therapy and have certain advantages and disadvantages. Extended daily dialysis (EDD) has emerged as an alternative to CRRT in the management of hemodynamically unstable AKI patients, mainly in developed countries.

**Objectives:**

We hypothesized that EDD is a safe option for AKI treatment and aimed to describe metabolic and fluid control of AKI patients undergoing EDD and identify complications and risk factors associated with death.

**Study Selection:**

This is an observational and retrospective study describing introduction of EDD at our institution. A total of 231 hemodynamically unstable AKI patients (noradrenalin dose between 0.3 and 1.0 ucg/kg/min) were assigned to 1367 EDD session. EDD consisted of 6–8 h of HD 6 days a week, with blood flow of 200 ml/min, dialysate flows of 300 ml/min.

**Data Synthesis:**

Mean age was 60.6±15.8 years, 97.4% of patients were in the intensive care unit, and sepsis was the main etiology of AKI (76.2). BUN and creatinine levels stabilized after four sessions at around 38 and 2.4 mg/dl, respectively. Fluid balance decreased progressively and stabilized around zero after five sessions. Weekly delivered Kt/V was 5.94±0.7. Hypotension and filter clotting occurred in 47.5 and 12.4% of treatment session, respectively. Regarding AKI outcome, 22.5% of patients presented renal function recovery, 5.6% of patients remained on dialysis after 30 days, and 71.9% of patients died. Age and focus abdominal sepsis were identified as risk factors for death. Urine output and negative fluid balance were identified as protective factors.

**Conclusions:**

EDD is effective for AKI patients, allowing adequate metabolic and fluid control. Age, focus abdominal sepsis, and lower urine output as well as positive fluid balance after two EDD sessions were associated significantly with death.

## Background

The high mortality rate among critically ill acute kidney injury (AKI) patients remains an unsolved problem in intensive care units (ICU) in spite of the considerable technological progress in renal replacement therapy (RRT) [1]–[3]. Dialytic management of these patients is difficult because of associated hemodynamic instability and multiple organ dysfunction, with mortality rates reaching 50–70% [4].

There is no consensus in literature on the best dialysis method and intermittent hemodialysis (IHD) and continuous renal replacement therapies (CRRT) have been used in AKI. Several studies have not revealed a definitive advantage in terms of patient survival for CRRT compared with IHD [5]–[10].

Both conventional IHD and CRRTs have certain advantages, but also several disadvantages. IHD is often complicated by hypotension and inadequate fluid removal, and CRRT by high cost of solutions and problems with anticoagulation. A hybrid therapy called sustained low efficiency dialysis (SLED) or extended dialysis (EDD) has emerged as an alternative to CRRT in the management of hemodynamically unstable patients with AKI, mainly in developed countries [11], [12].

The studies in the literature on EDD in AKI patients are few and involve a small number of patients [9], [13]–[16]. They have demonstrated that EDD is well tolerated in critically ill patients, with comparable ultrafiltration and solute removal to CRRT and peritoneal dialysis [13], [16].

This prospective study was designed to describe the introduction of EDD at our institution. We focused on metabolic and fluid control, complications and risk factors associated with death.

## Patients and Methods

### Study Population

This was an observational and retrospective study describing our experience of introducing EDD as a new HD modality in two Brazilian University Hospitals (Botucatu School of Medicine and Bauru State of Sao Paulo). In our units, conventional IHD and peritoneal dialysis had previously been the standard of care for AKI.

The protocol was approved by the institutional Ethical Committee (Comitê de ética em pesquisa da Faculdade de Medicina de Botucatu). Written informed consent was obtained from patients or their next of kin.

Patients were eligible for enrollment if they were critically ill, were 18 years of age or older, had clinical diagnosis of septic AKI or acute tubular necrosis (ATN) caused by ischemic or nephrotoxic injury. AKI was defined according to Acute Kidney Network Criteria [17].

Exclusion criteria were the patients with severe chronic kidney disease (basal creatinine higher than 4 mg/dl), previous chronic dialysis, kidney transplantation and using more than 1 mg/kg/min noradrenalin. These last patients were excluded because they could not tolerate net ultrafiltration (UF) 300–500 ml/h and because of that they were treated with CRRT.

Illness severity was determined according to Acute Tubular Necrosis–Index Specific Score (ATN-ISS) on the day of the first nephrology evaluation [18], [19]. Other variables included the etiology and causes of AKI, urine output at start of dialytic treatment, dialysis indication, number of dialysis sessions and need for mechanical ventilation were analyzed. Anthropometric measurements (weight, height, and body surface area) were obtained before dialysis. Body surface area was calculated from the DuBois formula [20]. Mobile patients were weighed on a digital scale, and weight of immobilized patients was obtained by a bed scale or calculated from two variable formulas [21].

Thereafter, patients treated with EDD were divided into two groups (survival and no survival) and then compared. The protocol was interrupted when there was partial recovery in renal function (urine output.>1000 ml/d) and progressive drop in creatinine (4 mg/dl) and BUN levels (50 mg/dl), more than 30 days of follow-up or death.

### Criteria for Dialysis and EDD Prescription

The indications for dialysis were uremic symptoms, BUN level >100 mg/dl (azotemia), fluid overload, oliguria, electrolyte imbalance (potassium>6 mEq l after clinical treatment), or acid-base refractory disturbances (bicarbonate<10 mEq/l_after reposition).

For practical reasons, it was decided that EDD would be carried out 6 to 8 h, six days a week (Monday–Saturday). Dialysis nurses and dialysis technical nursing were the responsible for EDD and operated the dialysis machines during all the treatment. A double lumen catheter for central venous access (jugular, subclavian, or femoral vein depending on the ease of access) was inserted blindly at the bedside, by nephrologists under local anesthesia. HD machine with volumetric control (*Fresenius 4008F* or *Gambro K200*) and cellulose acetate dialyzers (CA 150 or 170 with surface areas of 1.2 and 1.5 m^2^ respectively.) were used for each patient after the calculation of Kt/V. Blood flux was 200 mL/min and dialysate flux was 300 mL/min. Anticoagulation was achieved with unfractionated heparin (usually a 1000 U bolus followed by 500 U/h) or saline flushes of 100 ml given every 30 min if anticoagulation was contraindicated. If EDD was interrupted for procedures, it was restarted later attempting to complete 6–8 h of treatment.

UF was prescribed during dialysis treatment as per the daily requirements. UF was done at 300 mL/h to 500 mL/h and adjusted according to the alteration in hemodynamic parameters and fluid status of individual patients.

Bicarbonate (26 to 35 mEq/L), potassium (2 or 3 mEq/L), and sodium dialysate concentrations (142–148 meq/L) were adjusted according to individual requirements. Dialysate temperature was low (35.5°C) to prevent hypotension.

Hourly blood pressure monitoring was done during the procedures. Hypotension was defined as a single systolic blood pressure less than 90 mm Hg or a mean arterial pressure (MAP) less than 60 mm Hg.

Treatment duration, episodes of filter clotting and replacement, inotrope dose, and ultrafiltration rate were recorded at the end of each session. Post-treatment BUN levels were measured by the slow-flow method (with blood-pump speed reduced to 50 ml/minute). Blood samples were obtained from the arterial sampling port before the blood reached the dialyzer. HD adequacy was determined by using urea kinetic modeling based on Kt/V [22]. The delivered dose was determined by the single-pool Kt/V value, corrected for ultrafiltration but not for reappearance of urea nitrogen [22]. Blood urea, arterial blood pH, serum levels of bicarbonate, potassium, phosphate, urine output and fluid balance were recorded daily.

### Statistical Analysis

All analyses were performed according to the intention to-treat principle. Results are presented as mean and SD or median according to normality characteristics for each variable. T test was used to compare parametric variables between two groups and ANOVA followed by the Newman–Keuls test for multiple comparisons between groups. For nonparametric variables Mann-Whitney and Kruskal Wallis tests were used to compare two groups and multiple groups, respectively. For analysis of repeated measures, the Proc mixed program was used. Categorical variables were expressed as proportions and compared with the chi-squared test. Variables with significant univariate associations were candidates for multivariable analysis. Longitudinal multivariable logistic regression was performed using backward variable selection, with the exit criteria set at p<0.25. Variables not selected by the automated procedure were added back into the models individually to evaluate residual confounding, and covariate and propensity score adjustments were used to adjust for baseline differences. Statistical analyses were conducted using SAS version 9.2 for Windows (May of 2010).

Statistical significance was considered at a *p* value less than 0.05.

## Results

**Figure 1:**
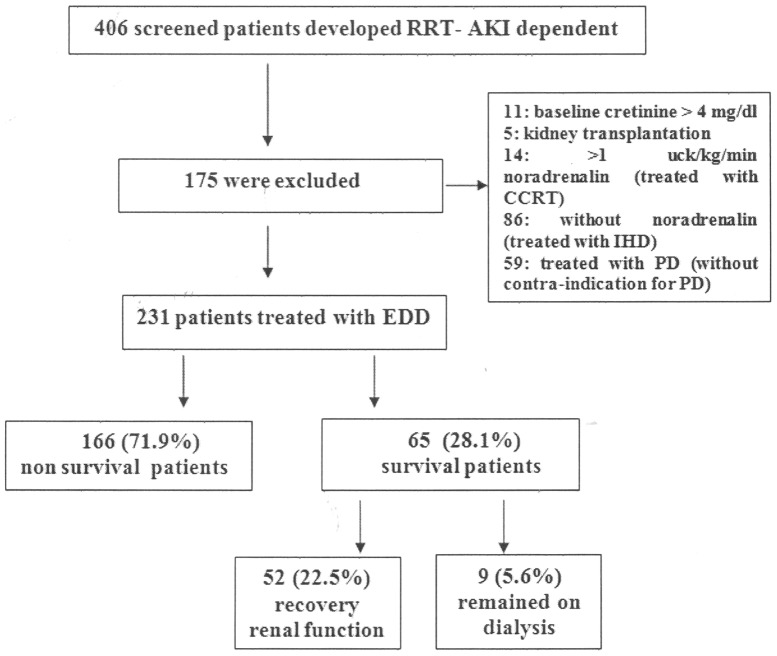
Inclusion and outcome of patients enrolled in the study.

During the study period (May 2009 to April 2012), a total of 406 patients were treated by dialysis: 247 by EDD (60.8%) and 86 by conventional IHD (21.2%), 14 by CRRT (3.5%) and 59 by high volume peritoneal dialysis (14.5%). Modality chosen was based on patients hemodynamic instability. PD was indicated when there was not contra-indication for its use (recent abdominal surgery, multiple abdominal surgeries, severe hyperkalemia with electrocardiogram changes, severe respiratory failure (FiO_2_<70%) and severe fluid overload). Conventional IHD was indicated for hemodynamically stable patients (without vasoactive drugs use). EDD was indicated when patients were using noradrenaline dose lower than 1 ucg/kg/min and CRRT when this dose was higher than 1 ucg/kg/min. Sixteen patients treated with EDD were withdrawn (6.5%) during the course of the study before final data analysis because had severe kidney disease (baseline creatine higher than 4 mg/dl) or kidney transplantation. Of the remaining 231 patients were treated with 1367 EDD sessions and included in the final analysis (figure 1).

The mean age was 60.6±15.8 years, 57% of patients were male, 50.5% of patients were Caucasian, and the mean patient weight was 75.6±10.1 kg (11.8% were obtained by a digital scale, 54.2% were obtained by bed scale, and 34% were calculated from two variable formulas. Most of the patients (97.4%) were in the ICU, 0.45±0.16 ucg/kg/min was the mean noradrenalin doses used, 92% were on mechanical ventilation; 79% of patients had low urine output and mean APACHE II and ATN-index specific scores (ISS) were 29.8±8.9 and 0.65±18, respectively. Sepsis was the main cause of AKI (76.2%) followed by ischemic ATN (21%). Oliguria or fluid overload was the main indication for dialysis (39.8%), followed by uremia or azotemia (23.8%), acidosis (17.3%) and hyperkalemia (12.9%). The median number of EDD sessions was 6, with a range of 4–10. Table 1 shows the clinical of AKI patients treated with EDD.

**Table 1:** Clinical characteristics of AKI patients treated with EDD.

Characteristics	N = 231
Male (%)	131 (57)
Age (year)	60.6±15.8
Caucasian (%)	116 (50.5)
Weight (kg)	75.6±10.1
ICU (%)	225 (97.4)
Noradrenalin dosis (ucg/kg/min)	29.8±8.9
Etiology of AKI (%)	
septic	177 (76.2)
ischaemic	50 (21.6)
Dialysis Indication (%)	
Oliguria/fluid overload	91 (39.8)
Uremia/azotemia	55 (23.8)
Acidosis	40 (17.3)
Hyperkalemia	30 (12.9)
Number of sessions	6(4–10)

– index score specific. UCI: unit care intensive; ATN-ISS: acute tubular necrosis

BUN and creatinine levels stabilized after four sessions at around 39 and 2.5 mg/dl, respectively and bicarbonate and pH levels stabilized after three sessions around 22.8 and 7.33 respectively. The mean UF remained stable during the treatment (2450±586 ml). Fluid balance decreased progressively and stabilized around 50 ml after five sessions. Weekly delivered Kt/V was 5.94±0.7. Table 2 shows metabolic control and fluid balance after EDD initiation.

**Table 2:** Serum BUN, Creatinine (Cr), Bicarbonate (Bic), pH, Potassium (K), Ultrafiltration (UF), Fluid Balance (FB) and delivered Kt/V of AKI patients after each session of extended dialy dialysis (EDD).

	Sessions							
	pre	1	2	3	4	5	6	p
	n = 247	n = 231	n = 210	n = 181	n = 168	n = 144	n = 122	
**BUN (mg/dl)**	88.9±31.9	62±22.6	51±13.2	45±17^a^	39±13^a^	38±11^a^	38±11.2^a^	<0.001
**Creatinine (mg/dl)**	4.2±1.4	3.9±.1.1	3.1±1.0	2.8±0.6	2.4±0.5^b^	2.3±0.5^b^	2.3±0.4^b^	<0.001
**Bic (mEq/L)**	17.5±4.7^c^	18.4±4.1	20.9±4.6	21.9±3.8	22.8±3.9	22.8±3.1	23.0±3.1	<0.001
pH	7.26±0.1c	7.28±0.1	7.30±0.2	7.30±0.2	7.32±0.2	7.33±0.2	7.32±0.2	<0.001
**K (mEq/L)**	5.1±0.77^c^	4.5±0.76	4.1±0.43	4.0±0.34	3.9±0.28	3.8±0.31	3.9±0.21	0.04
**Phosphate(mg/dl)**	5.96±0.4	5.58±0.3	5.3±0.3	5.1±0.2	5.12±0.2	4.33±0.3^d^	4.02±0.3^d^	<0.001
**UF (l/day)**	–	2250±410	2437.5±449	2567±251	2409±411	2550±418	2380±450	0.58
**FB (l/day)**	–	1120±212.4	512±38.6	314.4±57.7	200.1±31.8	51.9±10.7^d^	50.1±9.7^d^	0.03
**Urine output (ml)**	477.5±109	485.5±128	531.2±164	584.5±167	525.5±101	485.5±114	391.5±81	0.54
**Kt/v session**	–	0.98±0.12	1.02±0.13	1.01±0.11	0.97±0.10	0.99±0.12	1.01±0.11	0.73

^a^ significantly different from pre-dialysis values and after 1 EDD session.

^b^ significantly different from pre-dialysis values and after 1 EDD session.

^c^ significantly different from other sessions.

^d^ significantly different from after 1,2,3 and 4 EDD sessions.

Hypotension and filter clotting occurred in 47.5 and 12.4% of treatment session, respectively. Hypotension was transient and resolved with usual maneuvers employed during regular IHD session: discontinuation of UF or saline bolus infusion. Two hundred fifty one (18.4%) of EDD sessions required increase in inotropic suppor and EDD was interrupted because of ventricular tachycardia or increase of noradrenaline dose higher than 1 ucg/kg/min in on 19 occasions (1.4%). Hypophosphatemia occurred in 16% of patients.

Catheter related bacteremia developed in 5.1% of patients (8.1 episodes per 1.000 sessions-days). All patients had the catheter removed and the main etiologic agents were *Pseudomonas aeruginosa* and *MRSA*.

Concerning patient outcome, 22.5% of patients presented renal function recovery, 5.6% of patients remained on dialysis after 30 days, and 71.9% of patients died. Figure 1 shows patient outcome.

Afterward, patients were divided into survival (S) and no survival (NS) groups. The groups were similar in sex, sepsis as the main etiology of AKI (S = 68.1 vs. NS = 78.5%, p = 0.32) and fluid overload as the main indication of dialysis (S = 42.8% vs. NS = 45.7%, p = 0.21). There was no difference between S and NS patients in metabolic control and ultrafiltration rate. The groups had similar values of delivered Kt/V per week (S = 6.23 (5.89–6.26), vs. NS = 5.89 (5.83–6.01, p = 0.40) and rate of infectious and mechanical complications related to EDD (CRB was 6.0% in S vs 4.6% in NS, p = 0.57; hypotension and filter clotting occurred in S = 49.3% vs. 46.2% in NS, p = 0.79 and in S = 8.4% vs. 7.6% in NS, p = 0.59, respectively). Tables 3 and 4 show the metabolic control and parameters of dialysis adequacy.

**Table 3:** Acute kidney injury patients distribution treated with extended daily dialysis according to outcome and main clinical and laboratory characteristics.

	Non-survival	Survival	p
	(n = 166)	(n = 65)	
**Age (years)**	64 (52–71.5)	59 (51–66.3)	0.047
**APACHE II**	32.6 (30.6–34.1)	25.7 (21.9–26.2)	0.001
**ATN-ISS**	0.70 (0.61–0.78)	0.62 (0.48–0.67)	<0.001
**Male sex (%)**	56.6	57.6	0.83
**Main diagnosis (%)**			
Abdominal sepsis	40.9	12.3	<0.001
Pulmonary sepsis	28.7	30.6	0.94
Cardiovascular disease	21.2	36.7	0.046
Others*	10.2	20.4	0.07
**Etiology of AKI (%)**			
Septic ATN	78.3	69.2	0.12
Ischaemic ATN	20.6	32.7	0.16
**Mechanical ventilation (%)**	89.8	92.3	0.31
**Noradrenalin doses (ucg/kg/min)**	0.5 (0.3–0.6)	0.35 (0.2–0.5)	0.001
**Urine output (ml)**	306±61	521±239	0.01
**Dialysis indication(%)**			
Azotemia	22.5	24.5	0.81
Hyperkalemia	9.9	12.2	0.53
Fluid overload	45.7	42.8	0.59
Others**	21.9	20.5	0.85
**EDD complications** (%)			
CRB	6.0	4.6	0.57
Hypotension	46.2	49.6	0.79
Filter clotting	7.6	8.4	0.59
**Follow-up (days)**	11 (5–20)	14 (8–21)	0.02

others: liver diseases and post surgery.

others: acidosis, more than one indication.

**Table 4:** Acute kidney injury patient distribution treated with extended daily dialysis according to outcome and metabolic and fluid control.

	Non-survival	Survival	p
	(n = 166)	(n = 65)	
**BUN after (mg/dl)**			
1st session	65±22	59±21	0.34
2nd session	53±19	49±17	0.26
3rd session	49±17	43±19	0.34
4th session	42±18	37±12	0.49
5th session	40±12	36±11	0.57
**Creatinine after (mg/dl)**			
1st session	4.1±1.5	3.9±1.6	0.64
2nd session	3.6±1.2	3.2±1.4	0.71
3rd session	3.0±1.3	2.7±0.8	0.74
4th session	2.6±1.1	2.3±0.9	0.67
5th session	2.3±0.7	2.2±0.7	0.71
**Bicarbonate after (mEq/l)**			
1st session	16.4±4.7	18.1±4.9	0.51
2nd session	18.1±4.3	19.1±4.7	0.59
3rd session	21.2±3.5	20.9±4.5	0.69
4th session	22.5±3.4	22.8±4.4	0.71
5th session	22.8±3.1	22.7±4.1	0.87
**pH**			
1st session	7.25±0.11	7.26±0.16	0.89
2nd session	7.28±0.13	7.28±0.12	0.85
3rd session	7.30±0.19	7.31±0.18	0.59
4th session	7.31±0.11	7.30±0.11	0.61
5th session	7.33±0.18	7.32±0.13	0.57
**Phosphorus after (mg/dl)**			
	**Non-survival**	**Survival**	**p**
	**(n = 166)**	**(n = 65)**	
1st session	5.86±0.4	5.33±0.3	0.19
2nd session	5.67±0.3	5.22±0.2	0.15
3rd session	5.49±0.3	5.06±0.3	0.21
4th session	5.11±0.2	4.92±0.3	0.29
5th session	4.52±0.2	4.31±0.2	0.17
**UF after (ml)**			
1st session	2203±390	2423±325	0.57
2nd session	2415±243	2515±255	0.59
3rd session	2503±380	2485±260	0.63
4th session	2455±345	2560±480	0.68
5th session	2355±485	2555±445	0.61
**Fulid balance after (ml)**			
1st session	1203±390	823.2±325.9	0.37
2nd session	413.2±143	−26.25±16.8	0.19
3rd session	185.4±84.2	−78.2±6.4	0.03
4th session	111.7±48.1	−236±79	0.008
5th session	224.3±76.8	−452±116	0.001
**Delivered Kt/V**			
Per session	1.11±0.15	1.07±0.14	0.62
Weekly	6.65±1.2	6.42±1.7	0.57

There was significant difference between the groups in age (S = 59 (51–66.3) vs. NS = 64 (52–71.5), p<0.047), APACHE II (S = 25.7 (21.9–26.2) vs. NS = 32.6 (30.6–34.1), ATN-ISS (S = 0.62 (0.48–0.67) vs. NS = 0.70 (0.61–0.78), p<0.001), abdominal sepsis (S = 12.3% vs. NS = 40.9%, p<0.001), vasoactive drug doses (S = 0.35 (0.2–0.5) vs. NS = 0.5 (0.3–0.6), p<0.001) and follow up time (S = 14 (8–21) vs. NS = 11 (5–20), p = 0.02 (table 3). The two groups also differed in urine output and fluid balance (FB). FB was significantly lower after second EDD session and urine output was significantly higher in the S patients than in the NS group as table 4 shows. Nine factors met the criteria for inclusion in the multivariable analysis: age (OR = 1.027, 95% CI = 1.004–1.051, p = 0.02), APACHE II (OR = 1.44, 95% IC = 1.07–2.19), ATN-ISS (OR = 1.88, 95% CI = 1.11–2.49, p = 0.04), focus abdominal sepsis (OR = 1.3, 95% CI = 1.1–1.9, p = 0.01), cardiovascular disease (OR = 1.027, 95% CI = 1.02–1.05, p = 0.04) vasoactive drug doses (OR = 1.67, 95% CI = 1.1–1.9, p = 0.001), urine output (OR = 0.97, 95% CI = 0.95–0.99, p = 0.001), and FB after one EDD sessions (OR = 0.98, 95% CI = 0.97–0.99, p = 0.01). Age per 1 year (OR = 1.02, 95% CI = 1.01–1.06, p = 0.01), abdominal sepsis (OR = 1.31, 95% CI = 1.16–1.75, p = 0.002), urine output per 500 ml (OR = 0.98, 95% CI = 0.97–0.99, p = 0.03), and FB per –500 ml/day (OR = 1.39, 95% CI = 1.14–3.91, p = 0.001) were associated significantly with death, which is shown in Table 5.

**Table 5:** Association (with *p<0.25*) between multiple adjusted patient and extended daily dialysis characteristics and death.

Variables	OR (CI 95%)	p
**Age (per year)**	1.02(1.01–1.06)	0.01
**ATN-ISS**	1. 18 (0.99–2.11 )	0.07
**APACHE II**	1.07 (0.98–1.75)	0.06
**Noradrenalin doses**	1.32 (0.98– 1.64)	0.08
**Focus abdominal sepsis**	1.31 (1.16–1.75)	0.02
**Cardiovascular disease**	0.98 (0.97–1.01)	0.09
**Urine output (per 500 l/day)**	0.98 (0.97–0.99)	0.03
**FB (per - 500 ml/day)** *	0.91 (0.87–0.96)	0.002
**Follow-up (per 1 day)**	0.96 (0.92–1.08)	0.13

after first session.

## Discussion

In this observational and retrospective study, we evaluated the effective of EDD in unstable hemodynamically AKI patients in relation to metabolic and fluid control and identified risk factors associated with death. Two hundred thirty one patients were recruited. In line with other reports, encouraging results were observed for BUN, creatinine, bicarbonate and pH levels. There was a significant reduction in BUN and creatinine levels, with stabilization of BUN (<40 mg/dL), creatinine (<2.5 mg/dL) and bicarbonate levels (>22 mEq/L) after 4 sessions. In this study, delivered Kt/Vs per session and per week were around 1.0 and 5.9, respectively and they were enough to keep adequate metabolic control. Previous studies showed weekly Kt/V determined for EDD between 5.8 and 8.4 [15], [24]–[26]. Dialysis dose adequacy in AKI is a subject of controversy. Recently, several recent trials show that the relationship between dose of RRT and survival is not a linear one and weekly delivered Kt/V of 3.6 seems to be enough [26]–[29]. However, there is limitation of Kt/V as a marker of efficacy for this treatment method. A study by Elliot et al [30] showed that despite a comparable Kt/V the total solute removal for creatinine and urea increased with dialysis time from 4 over 6 to 8 hours, i.e. better solute removal despite identical Kt/V. This could be confirmed in a recent study by Schmidt et al [31], who compared pre and post dialysis uremic toxin concentrations and compared those to the total removal based on analysis of the spent collected dialysate.

Recent data demonstrate that neither the technique of renal replacement therapy (RRT) nor the dose of RRT had an impact on patient survival [24], [10], [27]–[29], [32]–[34]. In the meantime, the newer hybrid technique, EDD, which combines the hemodynamic stability of CRRT with a more favorably priced dialysis technique, has been introduced as a new cost-effective approach to the treatment of AKI in the ICU [6]–[15], [32], [34], mainly in developing countries.

There are few studies on EDD in AKI patients and most of them included a small number of patients or are review articles.

Previous prospective smaller investigations showed EDD was very well tolerated [11]–[16], [23], [24], [32]. However, the maintained hemodynamic stability may also be the result of the extended duration of the EDD treatment prescribed [12], [32]. In this study, hypotension was frequent (47.5% of treatment sessions) and it was transient and resolved with usual maneuvers employed during regular IHD session: discontinuation of UF or saline bolus infusion. Two hundred fifty one (18.4%) of EDD sessions required increase in inotropic support and EDD was interrupted because of ventricular tachycardia or increase of noradrenaline dose higher than 1 ucg/kg/min in on 19 occasions (1.4%). Probably, increasing the time of duration of dialysis session for 8 or 10 hours can be an alternative to decrease number of hypotension. Others suggestions would be start EDD with a low UF rate and increase it after 20–30 min to avoid initial blood pressure drop or treating many of these patients with CRRT.

This study showed that net UF values kept stable during the sessions around 2500 ml, not exceeding 500 ml/hour. However, FB decreased progressively session by session and stabilized around 50 ml/day after 5 sessions.

In our study, filter clotting occurred in 12.8% of treatment sessions and it was lower than observed in previous studies. Berbece et al [15] showed filter clotting in 18% of heparin treatments and 29% of heparin-free treatments. Hypophosphatemia occurred in 16% of patients, similar to that reported by Palevsky et al in 2008 [27].

A potentially complicating factor is infection related to catheter. CRB rate was 8.1 episodes per 1.000 CVC-days, higher than those reported in chronic patients using tunneled catheters, however similar to those shown in non-tunneled catheters [34]. All patients were treated with antibiotics and had their catheter removed and re-inserted in other site. There is no data about CRB in AKI patients treated by EDD to be compared to our results.

Concerning patient outcome, 22.5% of patients presented renal function recovery, 5.6% of patients remained on dialysis after 30 days, and 71.9% of patients died. In this study mortality rate was higher than that related in previous American and European studies, which showed in-hospital mortality rate of AKI patients treated with EDD ranged from 50 to 62% [6], [9], [13], [15], [24]. However, studies performed in developing countries as Brazil and India reported similar mortality rate [35]–[37].

There was no significant difference between the survival and no survival patients in relation to metallic control and delivered dialysis dose, in agreement with Palevsky et al [27], Bellomo et al [28] and Faulhaber-Walter R et al [33] in the trials ATN, RENAL and HANDOUT, respectively. Previous studies showed higher delivered clearance has been associated with higher rate hypophosphatemia ranging from 18% up to 66% of patients in the intensive-therapy arms of prospecitive randomized trials [27], [28] and hypophosphatemia alone has recently been shown to be associated with increased duration of mechanical ventilation and a higher all-cause in-hospital mortality and long-term mortality [38], [39]. In this study delivered clearance was similar between survival and no survival groups and probably because of that there was no difference in phosphate levels between the two groups and hypophosphatemia was not predictive for mortality.

No survival patients had clinical parameters and prognostic scores more severe than survival patients, as higher age, more abdominal sepsis, lower urine output, higher ATN-ISS and vasoactive drug dose. The two groups presented significant statistically difference in FB. After 2 EDD sessions, FB was statically lower in survival patients than in no survival group. Time of follow up time was also higher in survival patients.

These results are in agreement with previous studies that reported low urine output, fluid overload and sepsis are associated with worse prognostic of AKI patients [40]–[42]. Recent studies have showed that fluid overload is risk factor of death in critical patients. Clinical data show that positive fluid balance and oliguria can contribute negatively to prognosis lung, leading to increased time of invasive mechanical ventilation [43]–[45].

In multivariate analysis, age and abdominal sepsis were risk factors associated with death and urine output, and negative FB were protector factors associated with death.

There are several limitations of our study. First, given the observational nature of this study no conclusions may be reached on clinical outcomes (mortality or morbidity rates). Second, the very small numbers weakens the comparison between survivors and non-survivors and the exclusion of the sickest patients (14 patients receiveing noradrenaline dose hiher than 1 ucg/kg/min) can bias the study towards a benefit for EDD. Further analysis will be undertaken shortly with the results of this study, such as costs, evaluation of the catabolic state of patients, improvement in nutritional status after each dialysis session and patients and kidney survival curves. In addition, patients should be evaluated according to different levels of prognostic score in order to define in the same range of severity, patients in both groups showed similar changes.

Although these limitations, there were enough treatment days to permit useful data for the parameters of interest to us. It is the largest experience reported in literature on EDD in AKI patients (231 patients underwent 1367 EDD sessions). The findings of our study suggest that EDD may provide adequate treatment for the most AKI patients, achieving adequate metabolic and fluid control. However, hypotension was the most frequent complication related to this dialysis method and it does certainly not contribute to renal (or cardiac, brain, gut) functional recovery. Future work in this area should aim to clarify factors that inform decision-making around time of EDD modality. Maybe the increasing of dialysis session time for 10 or 12 hours can decrease the number of hypotension episodes. Others suggestion would be start EDD with a low UF rate and increase it after 20–30 min to avoid initial blood pressure drop or treating many of these patients with CRRT. We also have provided new information on the risk factors for death of patients treated with EDD as age, abdominal sepsis, low urine output and positive fluid balance after 2 sessions of EDD. Larger and trial studies will need to clarify the impact of EDD on patient survival and kidney function recovery.
